# Occurrence and Antibiotic Resistance of Mesophilic Aeromonas in Three Riverine Freshwaters of Marrakech, Morocco

**DOI:** 10.1100/tsw.2001.284

**Published:** 2001-12-01

**Authors:** Boujamaa Imziln

**Affiliations:** Laboratory of Microbiology, Department of Biology, Faculty of Sciences Semlalia, Road My Abdallah, P.O. Box 2390, Marrakech 40 000, Morocco

**Keywords:** Aeromonas, occurrence, antibiotic-resistance, antibiotics, pollution, sewage, arid climate, rivers, freshwaters

## Abstract

I n order to evaluate the impact of pollution and sewage on the occurrence and antibiotic resistance of mesophilic aeromonads in riverine freshwaters of Marrakech, samples were collected from three rivers (Oukaimeden, Ourika, and Tensift) upstream and downstream from the principal bordering villages. During a 2-year study, indicators of pollution increased dramatically in the downstream waters. Bacterial indicators (faecal coliforms and faecal streptococci) correlated with mesophilic aeromonads only in heavily polluted waters. In low and moderately polluted sources, densities of mesophilic aeromonads were independent of water quality indicators and did not correlate statistically with faecal indicators. Average counts of Aeromonas in low and heavily polluted waters were 2.5 × 103 and 2.1 × 106 colony forming units per 100 ml, respectively. The biochemical identification of 841 isolates indicated a predominance of A. caviae in heavily and moderately polluted water and sediment. A. hydrophila was dominant only in low polluted waters and when the temperature was below 12°C. High densities of A. sobria were found in low, moderately polluted, or cleaned waters and when the water temperature was above 18°C. All selected isolates (total = 841) were tested for antibiotic susceptibility against 21 antibiotics. Antibiotic resistance frequencies recorded were: ampicillin and amoxicillin, 100%; novobiocin, 96%; cefalotin, 81%; colistin, 72%; sulfamethoxazole, 40%; cefamandole, 37%; polymyxin B, 23%; trimethoprim, 17%; erythromycin, 15%; streptomycin, 8%; amoxicillin-clavulanate, 5%. Resistance to cefotaxime, kanamycin, gentamycin, chloramphenicol, tetracycline, oxytetracycline, nalidixic acid, rifampicin, or trimethoprim-sulfamethoxazole was found to be <5%. Antibiotic resistance rates did vary according to the source of a strain’s isolation, and high numbers of antibiotic resistant strains were recorded in polluted samples. Since no correlation between mesophilic aeromonads and conventional faecal pollution indicators was observed in low or moderately polluted waters, and since these freshwaters are used for domestic supply, we propose the use of mesophilic aeromonads as complementary water pollution indicators to ensure the safety of water.

### INTRODUCTION

During the last 20 years, special attention has been paid to mesophilic *Aeromonas* spp.[9,24,26]. Mesophilic aeromonads are ubiquitous in aquatic environments, and have been found in a wide variety of waters including both polluted and unpolluted waters[19,21]. These organisms have been frequently recognised as responsible for several diseases in humans that include diarrhoeal disease, extraintestinal infections, septicaemia, and wound infections[9,23,42]. The aquatic environments are the principal reservoir of mesophilic *Aeromonas* spp. and they are considered as the major source of infections[1]. The highest densities of mesophilic aeromonads were recorded from polluted waters and especially from sewage effluents with more than $10^8$ cfu/100 ml[20,32,34].

Several parts of Morocco, such as the Marrakech region, are subject to hazards of the arid climate. Such areas have very low annual rainfall (<242 mm) with a long period of drought (more than 7 months)[16]. The aridity of the climate makes water supply and its preservation a major concern. Marrakech is a wide continental area with natural water resources restricted to freshwaters. The supply of these resources depends on rainfall. Many rivers drain towards the Atlantic Ocean and during their passage, they provide water to irrigate agricultural land. In the valleys, the drained water also is used for domestic supply. On the banks of the major rivers in the area, rural (e.g., Oukaimeden and Ourika) and urban (Marrakech City) populations occur, and river waters are typically the main receptacle for pollutants that are from the bordering populations. Antibiotic-resistant bacteria are one of the pollutants that are abundant in the environment as a result of the increasing use of antibiotics in medical, veterinary, and agricultural practices[8,33].

In order to evaluate the water quality of some rivers in the Marrakech region, a study was carried out to investigate the occurrence of mesophilic *Aeromonas* spp. in three rivers (Oukaimeden, Ourika, and Tensift) of Morocco. The aim was to investigate the occurrence, concentration, diversity, and antibiotic resistance of different *Aeromonas* phenospecies and their correlation with water quality indicators. The general application of this work is in regard to identifying whether standard tests for faecal pollution of river water are adequate to identify the presence of *Aeromonas* bacteria.

### MATERIALS AND METHODS

#### Study Area and Sample Collection

The Oukaimeden, Ourika, and Tensift Rivers receive pollution from bordering populations of the Oukaimeden Village (31°12’ north 07°51’ west, 2600-m alt.), Ourika Village (31°20’ north, 07°24’ west, 1000-m alt.), and Marrakech City (31°39’ north, 8°01’ west, 380- to 440-m alt.). Wastes are discharged into the rivers without any treatment. Sampling stations in each river included one site upstream of the population source and one downstream (OK1 and OK2 in the Oukaimeden River, OR1 and OR2 in the Ourika River, TF1 and TF2 in the Tensift River) (Fig. 1). Water samples (1.5 l) were collected at all sites monthly from November 1998 to October 2000 using sterile glass bottles, and stored in cold boxes at 4°C until analysis in the laboratory within 8 h of collection. Environmental parameters measured on site included temperature, dissolved oxygen, and pH. Chemical (COD) and biological oxygen demand (BOD) and bacteriological determinations were performed in the laboratory.

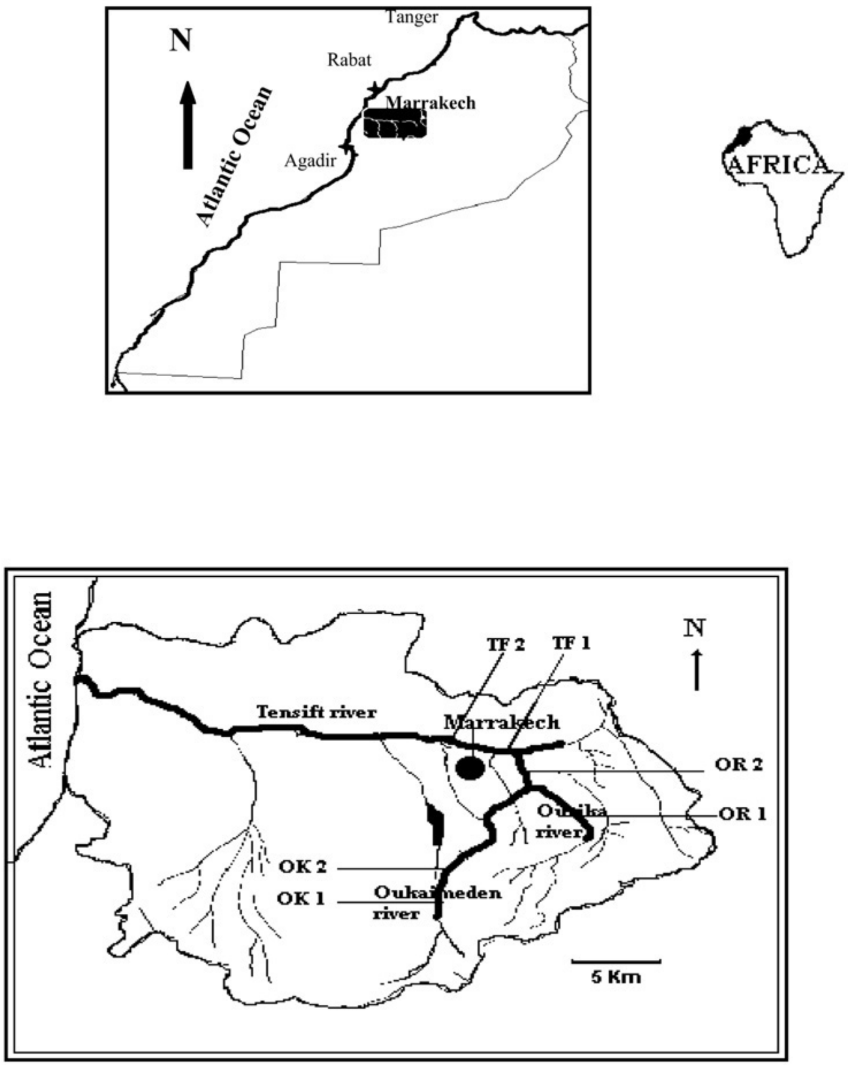

**FIGURE 1**. Map of Morocco indicating locations of sampling sites in the Oukaimeden, Ourika, and Tensift Rivers.

### Bacteriological Analysis

Samples were shaken for a period of 30 min and then diluted in sterile saline solution (0.85% [w/v] NaCl in demineralized water). Aliquots of 0.1 ml from samples of suitable dilutions were spread or poured onto specific media. Faecal coliforms were estimated using Tergito 7 Agar and incubated at 44.5°C for 24 h[27]. Faecal streptococci were enumerated on Bacto bile esculin azide agar as previously described[22], the incubation was made at 37°C for 24 h. Heterotrophic counts were estimated on plate count agar after incubation at 25°C for 1 week[17]. Mesophilic *Aeromonas* spp. were isolated and quantified on PADE agar as described earlier[19]. Inoculated plates were incubated at 37°C for 24 h. The pour plate method was used for quantification of faecal streptococci, while the spreading technique was used to estimate counts of other bacterial groups.

Typical *Aeromonas* colonies were collected and purified on CASO-Agar (Merck, 5458) plates and identified to phenospecies level by the method of Popoff[35] as recommended by Austin et al.[7] for environmental isolates.

### Antibiotic Susceptibility Testing

Antibiotic susceptibility of *Aeromonas* isolates was determined by the methods of Calomiris et al.[10]. Purified isolates were put by multipoint inoculation on Mueller-Hinton agar (Merck, 5437) plates supplemented with the following antibiotics (µg/ml): ampicillin, 10; amoxicillin, 25; amoxicillin-clavulanate, 120; cefalotin, 30; cefamandole, 30; cefotaxime, 30; streptomycin, 20; kanamycin, 20; gentamycin, 10; erythromycin, 15; chloramphenicol, 20; polymyxin B, 37.5 (300 units); tetracycline, 10; oxytetracycline, 10; colistin, 10; novobiocin, 25; nalidixic acid, 30; rifampicin, 30; trimethoprim, 5; sulfamethoxazole, 100; and trimethoprim-sulfamethoxazole, 1.25 + 23.75. These antibiotics were selected because they are widely used for the treatment of Gram-negative bacterial infections. After 24 h of incubation at 35°C, cultures on both Mueller-Hinton with and without antibiotics were compared, and strains were classified as sensitive or resistant.

### Data Analysis

All bacterial counts were $\log_{10}$ transformed before analysis. The statistical significance of differences in bacterial counts recorded for the different sources were evaluated using the nonparametric Wilcoxon signed rank test[39]. Correlation analyses were performed using the Pearson correlation test. To compare the level of antibiotic resistance of *Aeromonas* spp. isolated from the different samples, an antibiotic resistance index (ARI) was calculated according to Hinton and Linton[15] using the following formula:

$$ARI = x/ny$$

where x is the number of resistant determinants in a population with y individuals, and n is the number of antibiotics tested.

## RESULTS

### Mesophilic Aeromonas Distribution and Water Quality

During the 2-year study, the indicators of pollution increased dramatically downstream from the population sources (Table 1). Neither the pH nor the temperature changed significantly after riverine waters passed pollution sources. However, significant changes were recorded for dissolved oxygen, COD, and BOD. Water from downstream stations on all three rivers displayed higher faecal pollution than at upstream stations. Mesophilic aeromonads were recovered in all samples and their numbers ranged between 40 and $9 \times 10^5$ cfu 100 $ml^{-1}$ in samples from the Oukaimeden River, between $10^3$ and $4 \times 10^6$ cfu 100 $ml^{-1}$ in samples from the Ourika River, and between $2 \times 10^4$ and $2 \times 10^7$ cfu 100 $ml^{-1}$ in samples from the Tensift River. Comparison of *Aeromonas* counts between the upstream and the downstream stations indicated increases of more than 5-fold in the Oukaimeden River, more than 21-fold in the Ourika River, and more than 33-fold in the Tensift River. According to faecal pollution levels recorded at the six stations, samples were classified in three groups: slightly, moderately, and heavily polluted waters (Table 2).

**TABLE 1**
**Mean ± Standard Deviation Values of Physical, Chemical, and Bacteriological Data of 24 Riverine Water Samples at Each Station**

| Stations* | Temp. (°C) | pH | DO (mg/l) | BOD5 (mg/l) | COD (mg/l) | Total hetero-trophic** | Faecal coli-forms** | Faecal strepto-cocci** | Mesophilic *Aeromonas* spp.** |
|---|---|---|---|---|---|---|---|---|---|
| OK1 | 12.12 ± 3.79 | 7.41 ± 0.23 | 8.92 ± 1.30 | 14.74 ± 6.93 | 33.58 ± 14.84 | 5.74 ± 0.65 | 0.67 ± 0.68 | 2.07 ± 0.60 | 3.39 ± 0.70 |
| OK2 | 11.85 ± 4.92 | 7.44 ± 0.16 | 7.74 ± 1.21 | 32.75 ± 9.94 | 123.13 ± 54.56 | 5.73 ± 0.36 | 2.20 ± 0.47 | 2.61 ± 0.47 | 4.08 ± 0.89 |
| OR1 | 14.49 ± 4.02 | 7.79 ± 0.17 | 7.94 ± 0.85 | 14.01 ± 9.44 | 34.42 ± 18.66 | 5.72 ± 0.21 | 2.49 ± 0.59 | 2.66 ± 0.37 | 3.73 ± 0.54 |
| OR2 | 17.69 ± 4.96 | 8.12 ± 0.21 | 7.47 ± 0.54 | 71.04 ± 27.31 | 217.08 ± 100.04 | 6.92 ± 0.39 | 3.77 ± 0.67 | 3.91 ± 0.58 | 5.06 ± 0.87 |
| TF1 | 18.42 ± 5.25 | 7.79 ± 0.14 | 7.74 ± 0.46 | 65.25 ± 14.29 | 254.08 ± 50.63 | 6.43 ± 0.43 | 2.32 ± 0.52 | 2.46 ± 0.55 | 4.81 ± 0.30 |
| TF2 | 19.71 ± 6.07 | 7.60 ± 0.30 | 3.89 ± 1.64 | 234.46 ± 31.58 | 923.25 ± 163.46 | 8.68 ± 0.79 | 5.45 ± 1.01 | 4.81 ± 0.73 | 6.33 ± 0.56 |

*Note*: DO, dissolved oxygen; BOD5, 5-day biochemical oxygen demand; COD, chemical oxygen demand.

* Sampling sites: OK1, upstream site in the Oukaimeden River; OK2, downstream site in the Oukaimeden River; OR1, upstream site in the Ourika River; OR2, downstream site in the Ourika River; TF1, upstream site in the Tensift River; TF2, downstream site in the Tensift River.
** The results are expressed as geometric mean of log (cfu 100 $ml^{-1}$).

**TABLE 2**
**Bacteriological Counts from Each Source***

| Sampling site | Bacteriological parameters | GMEAN | AMEAN | MIN | MAX | STDEV |
|---|---|---|---|---|---|---|
| Slightly polluted OK1 (n = 24) | Heterotrophics | 5.71 | 5.74 | 5.08 | 7.75 | 0.65 |
| | *Aeromonas* | 3.31 | 3.39 | 1.61 | 4.42 | 0.70 |
| | Faecal coliforms | — | 0.67 | 0 | 2 | 0.68 |
| | Faecal streptococci | 1.97 | 2.07 | 1.04 | 3.11 | 0.60 |
| Moderately polluted (OK2, OR1, OR2, TF1) (n = 96) | Heterotrophics | 6.17 | 6.20 | 5.08 | 7.70 | 0.62 |
| | *Aeromonas* | 4.34 | 4.42 | 2.6 | 6.54 | 0.87 |
| | Faecal coliforms | 2.57 | 2.69 | 1.32 | 4.9 | 0.84 |
| | Faecal streptococci | 2.82 | 2.91 | 1.3 | 5.02 | 0.77 |
| Heavily polluted TF2 (n = 24) | Heterotrophics | 8.65 | 8.68 | 7.69 | 10.12 | 0.79 |
| | *Aeromonas* | 6.30 | 6.33 | 5.51 | 7.28 | 0.56 |
| | Faecal coliforms | 5.36 | 5.45 | 1.36 | 7.12 | 1.01 |
| | Faecal streptococci | 4.76 | 4.81 | 3.68 | 6.42 | 0.73 |

* The results are expressed in log scale as geometric mean (GMEAN), arithmetic mean (AMEAN), minimum (MIN), maximum (MAX), and standard deviation (SDEV). Bacterial group: cfu/100 ml.

Densities of mesophilic aeromonads in natural waters were found to be moderately correlated with the faecal pollution indicators (r < 0.50), in contrast with those observed from the heavily polluted waters (r > 0.85). A significant positive correlation with the heterotrophic populations was recorded only for the heavily polluted samples (r = 0.91). Similarly, a positive correlation with organic matter (expressed in COD or BOD) was observed for all systems. No correlations were found between concentrations of mesophilic aeromonads and pH or dissolved oxygen (Table 3). In slightly polluted waters, *A. hydrophila* was dominant (42%) relative to *A. caviae* (31%) and *Aeromonas* spp. (27%) (Table 4). In moderately and heavily polluted waters (except TF1 and TF2 sites), *A. caviae* (53 to 69%) was the most frequent species isolated. On the other hand, the percentage of *A. sobria* was clearly more important in Tensift River, where its incidence reached 45 and 39%, respectively, for TF1 and TF2 site (Table 4). Based on physical and chemical data obtained at the sampling sites and characterisation of 722 *Aeromonas* isolates collected, *A. sobria* was the most frequently encountered species in moderately polluted waters at a water temperature of >18°C. *A. caviae* was most abundant in waters immediately impacted by raw sewage.

**TABLE 3**
**Pearson Correlation Coefficient Between Mesophilic Aeromonads and Various Biotic and Abiotic Characteristics**

| Source | **Temp.** | **pH** | **DO** | BOD | **COD** | **Total Heterotrophic** | **Faecal Coliforms** | **Faecal Streptococci** |
|---|---|---|---|---|---|---|---|---|
| Low polluted | 0.69 | -0.05 | 0.09 | 0.56 | 0.58 | 0.12 | 0.40 | 0.46 |
| Moderately polluted | 0.73 | 0.41 | -0.42 | 0.74 | 0.78 | 0.56 | 0.45 | 0.49 |
| Heavily polluted | 0.86 | 0.30 | 0.18 | 0.49 | 0.65 | 0.91 | 0.92 | 0.86 |

**TABLE 4**
**Percentages of *Aeromonas* spp. at Each Station**

| **Station*** | **No**** | ***A. hydrophila*** | ***A. caviae*** | ***A. sobria*** | ***Aeromonas* spp.** |
|---|---|---|---|---|---|
| OK1 | 108 | 41.6 | 31.5 | 0 | 26.9 |
| OK2 | 107 | 23.4 | 62.6 | 0 | 14 |
| OR1 | 114 | 9.6 | 69.3 | 4.4 | 16.7 |
| OR2 | 125 | 29.6 | 52.8 | 8 | 9.6 |
| TF1 | 114 | 14.9 | 18.4 | 44.7 | 22 |
| TF2 | 154 | 9.1 | 30.5 | 39 | 21.4 |

* Abbreviations defined in Table 1.
** The number of strains isolated at each station.

**Table 5**
**Percent Antibiotic Resistance among *Aeromonas* spp. Isolated from the Riverine Samples**

| Tested antibiotics | *A. hydrophila* (n = 198) | *A. caviae* (n = 376) | *A. sobria* (n = 134) | *Aeromonas* spp. (others) (n = 133) | Total (n = 841) |
|---|---|---|---|---|---|
| Ampicillin | 100 | 100 | 100 | 100 | 100 |
| Amoxicillin | 100 | 100 | 100 | 100 | 100 |
| Amoxicillin-clavulanate | 0 | 10.9 | 0 | 3 | 5.4 |
| Cefalotin | 97.5 | 98.1 | 24.6 | 66.9 | 81.3 |
| Cefamandole | 68.2 | 33 | 9 | 30 | 37.0 |
| Cefotaxime | 13.1 | 0.2 | 0.7 | 5.3 | 4.2 |
| Streptomycin | 2.5 | 11.2 | 11.2 | 4.5 | 8.1 |
| Kanamycin | 3 | 1.9 | 9.7 | 0.7 | 3.2 |
| Gentamycin | 0 | 0 | 0 | 0.7 | 0.1 |
| Erythromycin | 32.8 | 12.8 | 2.2 | 6 | 14.7 |
| Chloramphenicol | 0.5 | 2.1 | 0.7 | 0.7 | 1.3 |
| Polymyxin B | 71.7 | 3.7 | 3 | 23.3 | 22.7 |
| Tetracycline | 1.5 | 2.1 | 0 | 0.7 | 1.4 |
| Oxytetracycline | 0.5 | 2.7 | 0.7 | 0.7 | 1.5 |
| Colistin | 92.9 | 61.7 | 67.9 | 72.9 | 71.8 |
| Novobiocin | 98 | 99.2 | 93.3 | 85 | 95.7 |
| Nalidixic acid | 0.5 | 0.3 | 0.7 | 0 | 0.4 |
| Rifampicin | 0.5 | 0 | 0 | 0 | 0.1 |
| Trimethoprim | 8 | 27.9 | 3.7 | 10.5 | 16.6 |
| Sulfamethoxazole | 38.4 | 46.8 | 23.9 | 36.8 | 39.6 |
| Trimethoprim-Sulfamethoxazole | 1 | 6.6 | 0 | 1.5 | 3.4 |
| ARI | 0.35 | 0.30 | 0.21 | 0.26 | 0.29 |

### Antibiotic Resistance of Riverine Mesophilic Aeromonas

All of the isolates (841 total) of *Aeromonas* were resistant to ampicillin and amoxicillin (Table 5). More than 95% of the isolates were resistant to novobiocin. Most of mesophilic *Aeromonas* isolates displayed resistance towards cefalotin (81%), colistin (71%), sulfamethoxazole (39%), and cefamandole (37%). Resistance to polymyxin B, trimethoprim, and erythromycin were 23, 17, and 15%, respectively. Fewer than 10% of the strains were resistant to streptomycin, amoxicillin-clavulanate, cefotaxime, kanamycin, chloramphenicol, tetracycline, oxytetracycline, or the association trimethoprim-sulfamethoxazole. Resistance to nalidixic acid, rifampicin, or gentamycin did not exceed 1%.

According to the ARI calculated for each *Aeromonas* spp. group, *A. sobria* (ARI = 0.21) had the lowest antibiotic resistance and *A. hydrophila* (ARI = 0.35) had the highest. The result for *A. sobria* is related to its low resistance to cefalotin, cefamandole, and erythromycin.

According to the calculated ARI at the different sources, there was an increase in the rates of resistant strains downstream from Oukaimeden Village and Marrakech City (Table 6). At Ourika River there was no difference between the ARI recorded for isolates collected from the two sampling sites. Mean values of the ARI calculated among mesophilic aeromonads isolated from riverine waters and sediments were similar and did not show any significant difference ($p > 0.05$) (Table 7).

### TABLE 6
## Percent Antibiotic Resistance among *Aeromonas*

| | Oukaimeden River | | | Ourika River | | | Tensift River | | |
|---|---|---|---|---|---|---|---|---|---|
| **Tested Antibiotic** | **Up-stream (n = 108)** | **Down-stream (n = 107)** | **Sediment (n = 55)** | **Up-stream (n = 114)** | **Down-stream (n = 25)** | **Sediment (n = 32)** | **Up-stream (n = 114)** | **Down-stream (n = 154)** | **Sediment (n = 32)** |
| Ampicillin | 100 | 100 | 100 | 100 | 100 | 100 | 100 | 100 | 100 |
| Amoxicillin | 100 | 100 | 100 | 100 | 100 | 100 | 100 | 100 | 100 |
| Amoxicillin-clavunalate | 7.41 | 6.54 | 7.3 | 4.39 | 4 | 6.3 | 5.26 | 3.40 | 6.3 |
| Cephalothin | 85.19 | 100 | 100 | 97.37 | 89.60 | 100 | 49.12 | 61.04 | 78.1 |
| Cephamondol | 35.19 | 26.17 | 36.4 | 35.09 | 42.40 | 12.5 | 34.21 | 34.42 | 53.1 |
| Cefotaxime | 1.85 | 7.48 | 12.7 | 2.63 | 0 | 0 | 3.51 | 4.55 | 12.5 |
| Streptomycin | 3.70 | 8.41 | 1.8 | 4.39 | 6.40 | 0 | 9.65 | 17.53 | 9.4 |
| Kanamycin | 0.93 | 5.61 | 0 | 0 | 5.60 | 0 | 2.63 | 5.84 | 6.3 |
| Gentamycin | 0 | 0 | 0 | 0 | 0 | 0 | 0 | 0 | 0 |
| Erythromycin | 26.85 | 12.15 | 32.7 | 2.63 | 7.20 | 31.3 | 7.02 | 15.58 | 25 |
| Chloramphen-icol | 0 | 2.80 | 0 | 1.75 | 0.80 | 0 | 0.88 | 2.60 | 3.1 |
| Plymyxin B | 44.44 | 25.23 | 56.4 | 12.28 | 0.80 | 56.3 | 25.44 | 9.74 | 37.5 |
| Tetracycline | 0 | 3.74 | 1.8 | 2.63 | 0 | 0 | 0.88 | 1.29 | 3.1 |
| Oxytetracycline | 0 | 1.87 | 0 | 2.63 | 0 | 0 | 0.88 | 3.89 | 3.1 |
| Colistine | 76.85 | 66.36 | 96.4 | 71.05 | 63.20 | 75 | 68.42 | 71.43 | 78.1 |
| Novobiocin | 86.11 | 100 | 100 | 97.37 | 98.40 | 100 | 94.74 | 93.51 | 100 |
| Nalidixic acid | 0 | 0.93 | 0 | 0 | 0.80 | 0 | 0 | 0.65 | 0 |
| Rifampicine | 0.93 | 1.87 | 0 | 0 | 0.80 | 0 | 0 | 0.65 | 0 |
| Trimethoprim | 5.56 | 19.63 | 18.2 | 23.68 | 14.40 | 18.7 | 13.16 | 19.48 | 18.8 |
| Sulphameth-oxazol | 35.19 | 46.73 | 41.8 | 33.33 | 39.20 | 37.5 | 40.35 | 42.86 | 34.4 |
| Trimethoprim-Sulphamethox-azol | 0.93 | 1.87 | 0 | 4.39 | 4.80 | 0 | 1.75 | 7.79 | 3.1 |
| **ARI** | 0.2816 | 0.3022 | 0.3359 | 0.2857 | 0.2824 | 0.3036 | 0.2596 | 0.2816 | 0.3199 |

### TABLE 7
## Mean Values of the ARI Calculated among Mesophilic *Aeromonas* spp. Isolated from Riverine Waters and Sediments

| ***Aeromonas* spp.** | **Riverine Waters** | **Riverine Sediments** |
|---|---|---|
| *A. hydrophila* | 0.34 | 0.37 |
| *A. caviae* | 0.30 | 0.28 |
| *A. sobria* | 0.21 | 0.20 |
| *Aeromonas* spp. (others) | 0.27 | 0.35 |
| Total | 0.28 | 0.31 |

### DISCUSSION

The aim of this study was to investigate the occurrence and antibiotic resistance of mesophilic *Aeromonas* in riverine freshwaters under the arid climate of Marrakech. The present work indicates that densities of mesophilic aeromonads in three rivers of Marrakech were not similar. The highest densities were recorded in the Tensift River which crossed Marrakech City. This river is exposed to excessive pollution that includes both liquid and solid wastes from human populations of Marrakech. The urban effluents are known to contain high levels of bacteria belonging to human and animal commensal flora. In our earlier works, we reported that mesophilic aeromonad densities in domestic raw sewage of Marrakech exceeded $10^{8-9}$ cfu 100 $ml^{-1}$[19,20]. The abundance of *Aeromonas* in the Tensift River therefore seems to be due to sewage pollution as well as the high concentration of organic compounds and nutrients. The lowest counts of mesophilic aeromonads were recorded in the Oukaimeden River, a high mountain river that is less exposed to pollution. In low and moderately polluted areas, there were no significant correlations between densities of aeromonads and faecal pollution indicators used in this study. In contrast, a positive correlation was noted between aeromonads and organic matter (expressed in COD or BOD). In heavily polluted sites a positive and significant correlation was noted between aeromonad densities and those of faecal coliforms. The positive correlation between the concentrations of aeromonads and faecal indicators on Tensift River indicates that these organisms have mainly the same origin.

Past studies have concluded that the concentration of mesophilic *Aeromonas* spp. is strongly correlated with common water quality parameters[5,6,38]. Others have not[28,29,37]. The diversity of results might be explained by the characteristics of individual studied sites (e.g., geographical situation, climate, water quality, human activities). It is difficult to isolate particular factors that control mesophilic aeromonad populations in aquatic ecosystems. The present study indicated that *A. hydrophila* dominated in low polluted sources. The dominance of *A. hydrophila* in low polluted river waters coincided with water temperatures <12°C. The species most frequently isolated from riverine water samples studied was *A. caviae*. This was the predominant species in sewage and waters receiving faecal pollution[4,14,19,20,31,32]. The percentage of this phenospecies diminished only in low or unpolluted waters. Indeed, some reports described the *A. caviae* phenospecies as most frequently isolated from human faeces[2,11]. The higher rates of *A. caviae* among mesophilic *Aeromonas* populations might be an indicator of a fresh faecal pollution of riverine waters. The third phenospecies, *A. sobria*, was the most frequently isolated biotype, at warm temperatures, in unpolluted waters, waters with low faecal pollution, and auto-treated polluted waters. This finding agrees with reports of Araujo et al.[4], and Rhodes and Kator[37]. In our previous works[18,20,22], *A. caviae*, the dominant phenospecies in both raw domestic sewage and stabilisation pond sediments, disappeared rapidly during treatment by stabilisation ponds under the arid climate of Marrakech and in warm periods. At the exit of the treatment plants, the mesophilic aeromonad group was dominated by the *A. sobria* phenospecies. This is in agreement with other studies[31,40]. When we compare the survival ability of mesophilic aeromonads in stabilisation pond effluents, our results indicate that *A. sobria* strains survived more than *A. caviae*. *A. caviae* strains seemed to be more susceptible to biotic and abiotic factors[22]. Such findings may indicate a possibly improved survival and/or multiplication of *A. sobria* in treated polluted waters especially in warm periods.

As previously noted[14,18], all isolates of mesophilic aeromonads from rivers were found to be resistant to ampicillin and amoxicillin, and just a few ( <5%) strains were found to be susceptible to novobiocin. Among the 841 isolates studied, and according to the calculated ARI, *A. sobria* strains were most susceptible to antibiotics used in this study ($p < 0.05$). The most striking difference among the three phenospecies was in their resistance to cefalotin followed by cefamandole, erythromycin, and sulfamethoxazole. The difference in the distribution of susceptibility to cefalotin among aeromonads species was described previously[14,18,41].

In the present work, and except for some antibiotics that are known to be inactive against aeromonad bacteria (penicillins, first and second generation of cefalosporins, novobiocin, polypeptides), more than 90% of tested isolates were sensitive to the remaining antibiotics used in this study. This finding agrees with results of several authors who reported a strong susceptibility of aeromonads to chloramphenicol, kanamycin, gentamycin, tetracycline, oxytetracycline, nail-dixic acid, trimethoprim-sulfamethoxazole, and cefotaxime[25,30]. In contrast, other authors have reported different results concerning the percentage of antibiotic resistant aeromonads[3,12,13,36]. These differences in literature results may be related to the technique of isolation, the sources of isolation, number of tested strains in each study, the proportion of different species composing the mesophilic group, and the frequency of use of antibiotic drugs in specific geographic areas.

The impact of human activities and pollution on the distribution of antibiotic-resistant *Aeromonas* was investigated, and according to the obtained values of the ARI, there is an increased level of acquired resistance to antibiotics among mesophilic aeromonads from the upstream to downstream stations (especially at the Oukaimeden and Tensift Rivers). Similarly, when we compared the occurrence of antibiotic resistant strains at each station, downstream stations had higher quantities than upstream sites. Since the domestic and urban effluents are known to contain high levels of antibiotics and antibiotic-resistant bacteria belonging to human and animal commensal flora, discharge of sewage into rivers resulted in the increase of resistant strains of natural riverine bacteria. To avoid such results, pretreatment of sewage is strongly recommended.

### ACKNOWLEDGEMENTS

This work was supported by an International Foundation for Science (IFS) grant number A/2774-1 awarded to the author.
